# Radiomics for Gleason Score Detection through Deep Learning

**DOI:** 10.3390/s20185411

**Published:** 2020-09-21

**Authors:** Luca Brunese, Francesco Mercaldo, Alfonso Reginelli, Antonella Santone

**Affiliations:** 1 Department of Medicine and Health Sciences “Vincenzo Tiberio”, University of Molise, 86100 Campobasso, Italy; luca.brunese@unimol.it (L.B.); antonella.santone@unimol.it (A.S.); 2 Institute for Informatics and Telematics, National Research Council of Italy, 56121 Pisa, Italy; 3 Department of Precision Medicine, University of Campania “Luigi Vanvitelli”, 80100 Napoli, Italy; alfonso.reginelli@unicampania.it

**Keywords:** prostate, cancer, radiomic, deep learning

## Abstract

Prostate cancer is classified into different stages, each stage is related to a different Gleason score. The labeling of a diagnosed prostate cancer is a task usually performed by radiologists. In this paper we propose a deep architecture, based on several convolutional layers, aimed to automatically assign the Gleason score to Magnetic Resonance Imaging (MRI) under analysis. We exploit a set of 71 radiomic features belonging to five categories: First Order, Shape, Gray Level Co-occurrence Matrix, Gray Level Run Length Matrix and Gray Level Size Zone Matrix. The radiomic features are gathered directly from segmented MRIs using two free-available dataset for research purpose obtained from different institutions. The results, obtained in terms of accuracy, are promising: they are ranging between 0.96 and 0.98 for Gleason score prediction.

## 1. Introduction

According to the American Cancer Society, one in nine men will be diagnosed with prostate cancer during their lifetime: estimations about prostate cancers in 2018 report about 164,690 new cases and 29,430 deaths related to prostate cancer (https://www.cancer.org/cancer/prostate-cancer/about/key-statistics.html).

After breast and lung cancer, prostate one is the third most common cancer in America. In Italy, prostate cancer is the most common cancer that hits men, with an incidence of 12%, surpassing lung cancer which is around 10%. Every year in Italy 42,804 prostate cancers are registered with 9070 fatalities (http://www.prostatecancerprevention.net/index.php?p=prostate-cancer). Of prostate cancer, 99.9% of cases are labeled as adenocarcinomas but usually, the cancer is classified into different stages depending on the cancer progression. For instance, localized cancer is classified as stages 1 and 2 (representing the 79% of prostate cancers), while stages 3 and 4 are related to regional prostate cancer and they represent the 12% of prostate cancers. Prostate cancers that progressed far beyond the prostate belong to the 4th stage. Distant prostate cancer is contained in the rest of stage 4 that has progressed far beyond the prostate, and makes up 5% of cases, while the remaining 4% of cases are unknown or unstaged (https://prostatecancer.net/prostate-cancer-statistics/).

With the aim to provide a rank related to the cancer aggressiveness, each prostate cancer diagnosis is labeled with the Gleason score.

Considering that prostate cancers are often composed of several cancerous cells with different grades, for each subject two grades are assigned. The primary one is related to the largest area of the cancer, while the second one is assigned to describe cells to the second largest area after the first one: this is the reason why the Gleason score is expressed using two numbers. For instance, whether the Gleason score is equal to 3 + 4, the largest cancer area obtained a grade equal to 3 and the largest section after the first one obtained 4 as the grade. A diagnosed prostate cancer can be marked with one of the following Gleason scores (according to its extension):

- 3 + 3 = 6: the attribution to this category is symptomatic of there being individual discrete well-formed glands (this category is representing the less aggressive one);
- 3 + 4 = 7: the cancer is mainly composed of well-formed glands, but a small component of poorly formed, fused and or cribriform glands appears;
- 4 + 3 = 7: the cancer is mainly composed of poorly formed, fused and/or cribriform glands with fewer well-formed glands;
- 4 + 4 = 8: when a cancer is labeled as belonging to this category, it is usually composed only of poorly formed, fused and/or cribriform glands.

It is a crucial task to label a diagnosed prostate cancer with the correct Gleason score.

Radiomic is a new emerging field in which medical images, for instance, Magnetic Resonance Imaging (MRIs) are converted into numbers. Radiomic has the potential to uncover disease characteristics that fail to be recognized by the naked human eye. In this way it is possible to provide valuable information for personalized therapy. Radiomic features are obtained directly from magnetic resonance images and not from tissue (for this reason an invasive biopsy is not necessary to obtain the radiomic values). Moreover, as demonstrated in current literature [1,2,3] radiomic features are able to provide information about the gray-level patterns and their associations within a region of interest and, for this reason, can be employed to discriminate between cancerous and healthy areas. In fact, the radiomic feature analysis has enabled insights into the design of innovative prognostic imaging biomarkers, resulting in a better understanding of cancer and the development of computer-aided diagnosis solutions.

Deep learning methods demonstrated the ability to learn feature representations automatically from data under analysis [4]. These features are included in hidden layers of neural networks [5]. In this context, deep learning-based networks can be exploited in order to build predictive or prognostic non-invasive biomarkers from radiomic characteristics [6]. We exploit convolutional neural networks (CNN) considering that they have demonstrated to have good prediction performances in the detection of simple patterns within data, which will then be used to form more complex patterns within higher layers [7]. In detail, one-dimensional CNNs demonstrated to be very effective when we have to derive interesting features from shorter (i.e., fixed-length) segments [8,9,10].

Starting from these considerations, with the aim to support radiologists and doctors in timely diagnosis, in this paper we propose a deep learning-based method able to detect the Gleason score for prostate cancer directly from MRIs.

More precisely, we exploit a set of 71 radiomic features, which availability is enabling the extraction of knowledge directly from MRIs. In order to represent MRIs using numeric vectors, we consider radiomic features belonging to several categories: First Order, Shape, Gray Level Co-occurrence Matrix, Gray Level Run Length Matrix, Gray Level Size Zone Matrix.

The radiomic feature categories are considered as inputs to the designed deep learning network aimed to discriminate between different Gleason score prostate cancers. Using the designed network we built a model able to abstract several types of prostate cancers. Once the model is built from the radiomic features directly gathered from MRIs, we evaluate the effectiveness of the proposed method in the Gleason score prostate cancer detection using MRIs not included in the data considered to generate the model.

Below are summarized the distinctive points of the following contribution:

- we propose a method aimed to automatically discriminate between different prostate cancers and healthy cases;
- we consider a set of 71 radiomic features obtainable directly from MRIs;
- we designed and implemented a deep architecture based on convolutional layers aimed to demonstrate the effectiveness of the proposed feature set in Gleason score detection;
- we cross-evaluated the proposed method using real-world MRIs for research purposes obtained from three datasets freely available for research purposes. We consider freely available datasets for easy result replication;
- we compare the proposed method with supervised machine learning algorithms and with the Embedded Localisation Features technique with the aim to demonstrate the effectiveness of the designed deep learning network;
- to provide a diagnostic tool useful for radiologists, we present the obtained results also at a patient-grain.

The paper proceeds as follows: Section 2 describes the radiomic features, the classification approach, the deep learning network and the designed experiment to evaluate the effectiveness of the proposed method. The results of the experiment are provided in Section 3, Section 4 provides a reasoned discussion about the current literature about prostate cancer detection, while in Section 5 conclusions and future research lines are drawn.

## 2. Method

In this section, we describe the proposed method aimed to automatically discriminate between different Gleason score prostate cancers and healthy patients using a deep learning architecture. First, we introduce the evaluated radiomic features for Gleason score prediction, and in the next subsections we respectively describe the classification approach and the deep neural network we designed.

### 2.1. The Radiomic Feature Set

Radiomic features demonstrated the ability to exhibit disease characteristics failing to be understood by the naked eye [11]. For this reason, we considered 71 different radiomic features belonging to five different categories: First Order, Shape, Gray Level Co-occurrence Matrix (GLCM), Gray Level Run Length Matrix (GLRLM) and Gray Level Size Zone Matrix (GLSZM). The full list of the features considered is available in reference [12] with the detail about the category and a brief description for each radiomic feature.

More details about the considered features can be found in [12,13,14,15].

### 2.2. The Classification Approach

We consider supervised machine learning techniques. In order to build models, this kind of algorithm requires two main steps: Training and Testing.

**Figure 1:**
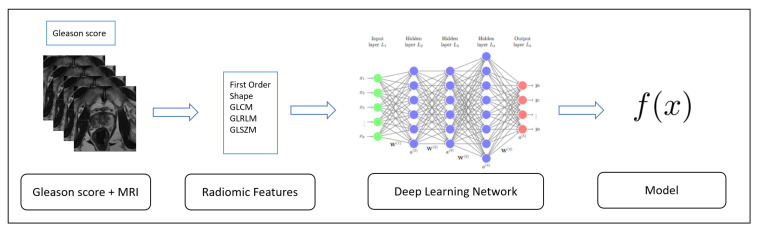
Training step aimed to build from labeled MRIs (i.e., MRIs with the detail about the Gleason scores).

The first one, the Training, is depicted in Figure 1: starting from the data (in this case the radiomic features gathered from MRIs), these algorithms generate an inferred function, aimed to discriminate between several Gleason scores.

In this study, the classes to discriminate are the ones related to Gleason scores (i.e., 3 + 3, 3 + 4, 4 + 3 and 4 + 4) and the one related to healthy patients (i.e., normal) described in the Introduction Section. We consider (as explained in the evaluation section) three freely available datasets related to different Gleason scores of prostate cancers and healthy patients, widespread for research purposes. The radiomic features considered are extracted on the ROI areas identified by radiologists.

**Figure 2:**
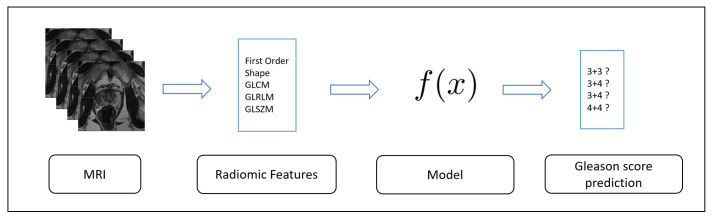
Testing step i.e., evaluation of the model built in training step through unseen MRIs to predict the Gleason scores.

The second step is related to the evaluation of the effectiveness of the model previously built in the detection of never seen prostate cancers MRI: the Testing one, is depicted in Figure 2.

In the evaluation, cross-validation is considered: basically, the full dataset is split into different five parts and we consider the first part as a training dataset and the second one as a testing dataset. This process is repeated five times, employing different MRIs with the aim to evaluate the full dataset.

### 2.3. The Deep Neural Network

The deep neural network we propose in this paper to automatically detect the Gleason score and healthy patients in MRIs considers Convolutional Neural Networks (CNN), a class of deep neural networks, largely applied to analyzing images. Convolutional layers use a convolution operation i.e., an operation between two functions of a variable that consists of integrating the product between the first and second translations of a certain value.

We consider several convolution layers in the designed neural network: as a matter of fact, usually, the first layer is able to detect abstract concepts while the deeper layers are aimed to learn less abstract concepts.

The neural network we designed consists of several one-dimesional (1D) convolutional layers, that have demonstrated to be useful in inferring interesting characteristics [4]. These characteristics related to 1D convolutional layers are matching the radiomic data we want to analyze. As a matter of fact, the position of the features in the dataset is not relevant, furthermore considering that we extract 71 features the considered sequence is not long and it is fixed length (in fact all the analyzed features are extracted from each MRI).

**Figure 3:**
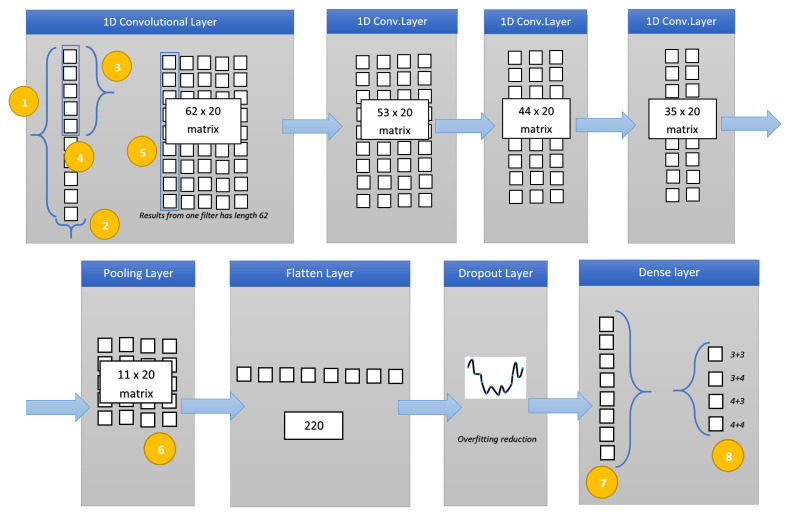
The designed deep neural network for Gleason score and healthy patients prediction.

In Figure 3 the designed deep neural network for Gleason score detection and healthy patients is depicted.

The deep network is composed of four 1D convolutional layers, one pooling layer, one flatten layer, one dropout layer and one dense layer: a total of eight hidden layers. In addition we have to consider two additional layers: the input layer (with 71 features as input i.e., 71 input neurons) and the output layer (with four neurons, one for each class to predict), in total, the deep neural network consists of 10 layers.

Below we describe in detail each layer:

- Input Layer: from each MRI we extract the 71 considered radiomic features. Each analyzed MRI is represented as a vector of 71 elements i.e., 71 neurons. This is the “height” of the dataset i.e., the length of one instance (i.e., an MRI) which is fed into the network (localized by the yellow point n. 1 in Figure 3). The yellow point n. 2 identifies the “width” of the instance which is fed into the network. In our case, this parameter is equal to 1 (the instance related to the MRI is modeled as a vector), the width is also referred to as the depth;
- 1D Convolution Layer: the first 1D convolutional layer. The yellow point n. 3 identifies the kernel size i.e., the size/height of the sliding windows that convolves across the data. It is also referred to as kernel size or filter length. We set this parameter equal to 10. With a height of 71 and a kernel size of 10, the window will slide through the data for 62 steps (71−10+1). The yellow point n. 4 identifies the filters i.e., how many sliding windows will run through the data. We set this parameter as 20, and in this way, 20 different features can be detected in this layer (also called feature detectors). This is the reason why this operation is results in a 62 × 20 output matrix: this initial layer will learn the basic features. The considered activation function is relu (also for the others considered convolutional layers of the network), because usually models with the relu activation function neurons converge much faster than neurons with other activation functions. Basically, the relu function gives an output *x* if *x* is positive and 0 otherwise (the choice of the activation function is a parameter chosen by the network designer);
- 1D Convolution Layer: the second 1D convolutional layer. We set the kernel size as 10 and the filters as 20: considering that the input matrix belonging to the first convolutional layer is 62 × 20, the output of this layer is a 53 × 20 matrix (62−10+1).
- 1D Convolution Layer: the third 1D convolutional layer. In this layer, we set the kernel size as 10 and the filters as 20. The input from the previous convolution layer is a 53 × 20 matrix, for this reason the output is resulting of a 44 × 20 matrix (53−10+1);
- 1D Convolution Layer: the last 1D convolutional layer. It considers as the input from the third convolution layer a 44 × 20 matrix and, considering that, as in the previous layers, we set the kernel size as 10 and the filters as 20, it outputs a 35 × 20 matrix (44−10+1);
- Pooling Layer: this kind of layer is usually considered after CNN layers with the aim to reduce the complexity. It is aimed to decrease the spatial dimensions. This step will prevent the overfitting of learned features by gathering the maximum value of multiple features using a sliding windows approach (yellow point n. 6 in Figure 3). In detail we consider a max-pooling layer, which slides a window in the same way as a normal convolution, and obtains the biggest value as the output (for instance from the [2, 4][5, 6] matrix the result will be [6] applying the max pooling with a size equal to 2). In the designed network, we consider a size equal to 3: the input 35 × 20 input matrix from the fourth convolutional layer is transformed in an 11 × 20 matrix. The output matrix size of this layer is a third of the input matrix;
- Flatten Layer: this layer is aimed to flat the layer input. As an example, a rows × columns matrix is flattened to a simple vector output of rows * columns shape. In this case the Flatten layer input is the 11 × 20 matrix, for this reason the output is a vector of 220 elements;
- Dropout Layer: we introduce another layer considered to prevent overfitting. This layer is aimed at randomly selecting neurons that are excluded in the training. In this way, their contribution is temporally avoided on the forward pass and, for this reason, any weight updates are not applied to the neuron on the backward pass. This improves generalization because we are forcing to learn, with different neurons, the same “concept”. We set the dropout parameter as 0.5:50% of neurons will be deactivated. Usually using the dropout layer, worse performances are obtained, but the aim is to trade training performance to obtain more generalization and the network will become less sensitive to smaller variations in the data;
- Dense Layer: this layer reduces the vector of height 220 to a vector of 5 elements (since we have to predict four different classes Gleason score based: 3 + 3, 3 + 3, 4 + 3 and 4 + 4 and the normal class i.e., the class related to healthy patients). This reduction is performed by matrix multiplication. We choose softmax as an activation function: it takes an un-normalized vector, and normalizes it into a probability distribution, it is usually considered for multiclass classification problems (yellow point n. 7 in Figure 3). The output will represent the probability for each of the four classes.
- Output Layer: the final output layer consists of five neurons (one for each class we have to represent) with included probability for each class. The neuron with the higher probability will be selected as a result of the prediction (yellow point n. 8 in Figure 3).

As a loss function, we consider the categorical cross-entropy, a loss function typically considered in multi-class classification tasks, while as an optimizer we exploit Adam, an adaptive learning rate optimization algorithm specifically designed for deep neural networks training. We resort to the Adam optimizer because empirical results show that this optimizer works well in practice and compares favorably to other stochastic optimization methods [16,17].

We designed an experiment to investigate whether the considered radiomic feature can be useful to discriminate between different Gleason scores and healthy patients.

The idea is to validate whether the considered radiomic features are able to predict the Gleason score or from an unseen MRI. The classification is carried out by using the deep neural network described in the previous section, with the 71 radiomic features as input neurons.

We design an experiment composed of three phases: the first one is a descriptive statistics comparison; the second one is represented by the hypotheses testing; and the last one is a classification devoted to understanding if the radiomic features are able to correctly discriminate between different unseen prostate cancer MRIs.

To compare the MRIs population through descriptive statistics we consider the boxplot, while for the hypotheses testing, the following null hypothesis is considered:

$H_{0}$: ‘MRIs prostate tumors labeled with different Gleason scores exhibit similar values for the exploited radiomic features’.

The Wald–Wolfowitz (with the p-level fixed to 0.05) and the Mann–Whitney Test (with the p-level fixed to 0.05) were considered for evaluating the null hypothesis.

With regards to the classification analysis, we implemented the designed deep neural network using the Python programming language with Keras, a library to work with deep learning network, able to run on the top of TensorFlow, an open-source software library for high performance numerical computation. The radiomic features have been extracted using PyRadiomics (https://pyradiomics.readthedocs.io/en/latest/), a Python package able to extract a set of radiomic features from MRIs. There is no specific meaning in the order of the features: as a matter of fact, changing the order of the feature does not change the results we obtain. When the developer chooses a feature order, this order must be respected (coherently with the model-building task of the classification approach) i.e., the order of the feature vector must be the same for each analyzed slice: one of the tasks implemented in the script we developed for radiomic feature extraction is the feature vector storing, performed in the same order for all the analyzed slices (the order we considered is the same appearing in the [12] in the radiomic feature list). Clearly, different order of the features exactly produces the same results.

**Figure 4:**
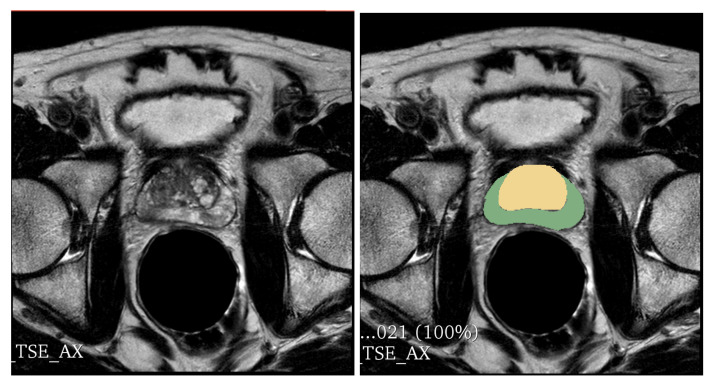
MRI slices related to a patient affected by 4 + 3 Gleason score prostate cancer.

Two prostate cancer public data-sets freely available for research purposes (https://wiki.cancerimagingarchive.net/display/Public/PROSTATE-DIAGNOSIS, https://wiki.cancerimagingarchive.net/display/Public/Prostate+Fused) are considered to evaluate the effectiveness of the proposed method. The first one is composed of 824 slices belonging to 36 patients while the second one is composed of 676 slices belonging to 26 patients, acquired using a 1.5 T Philips Achieva. The datasets are composed of prostate cancer T2-weighted MRIs acquired using a 1.5 T Philips Achieva by combined surface and endorectal coil, including dynamic contrast-enhanced images obtained prior to, during and after I.V. administration of 0.1 mmol/kg bodyweight of Gadolinium-DTPA (pentetic acid). The data-sets were collected from different institutions: the first one is from the Boston Medical Center, Boston, Massachusetts, USA and the second one from the National Cancer Institute, Bethesda, Maryland, USA. The considered MRIs contain the segmentation (i.e., the ROI) provided by radiologists and doctors, pathology biopsy and excised gland tissue reports. Furthermore, there is also the MRI radiology report. We consider different datasets belonging to different institutions (with the ROIs marked by different radiologists) to train the model using a dataset and perform the evaluation using the second independent dataset, but also with the aim to minimize the impact of the ROI selection. Figure 4 shows an example of slices related to a 4 + 3 Gleason score: the left slices are without the ROI and the lesion is visible, the right one with the ROI. Considering that each prostate cancer grade is composed of two values i.e., the cancer grade of the main mass and the cancer grade of the second largest mass, and for each mass there is an ROI we combine these two ROIs to compute the radiomic features. As a matter of fact, in this paper the idea is to detect the prostate cancer grade and not only the grade of the main mass or to the second one, for this reason we resort to a combined ROI for radiomic feature extraction. Moreover, to evaluate the effectiveness of the proposed deep learning network we take into account an additional data-set composed of normal cases. The normal cases data-set is freely available for research purposes (https://i2cvb.github.io/#prostate-data) and contains 300 MRIs belonging to 10 different healthy patients (30 slices for patient) [18,19].

## 3. Results

In this section, we present the result of the experimental analysis in terms of descriptive statistics, hypotheses testing and classification analysis.

### 3.1. Descriptive Statistics

**Figure 5:**
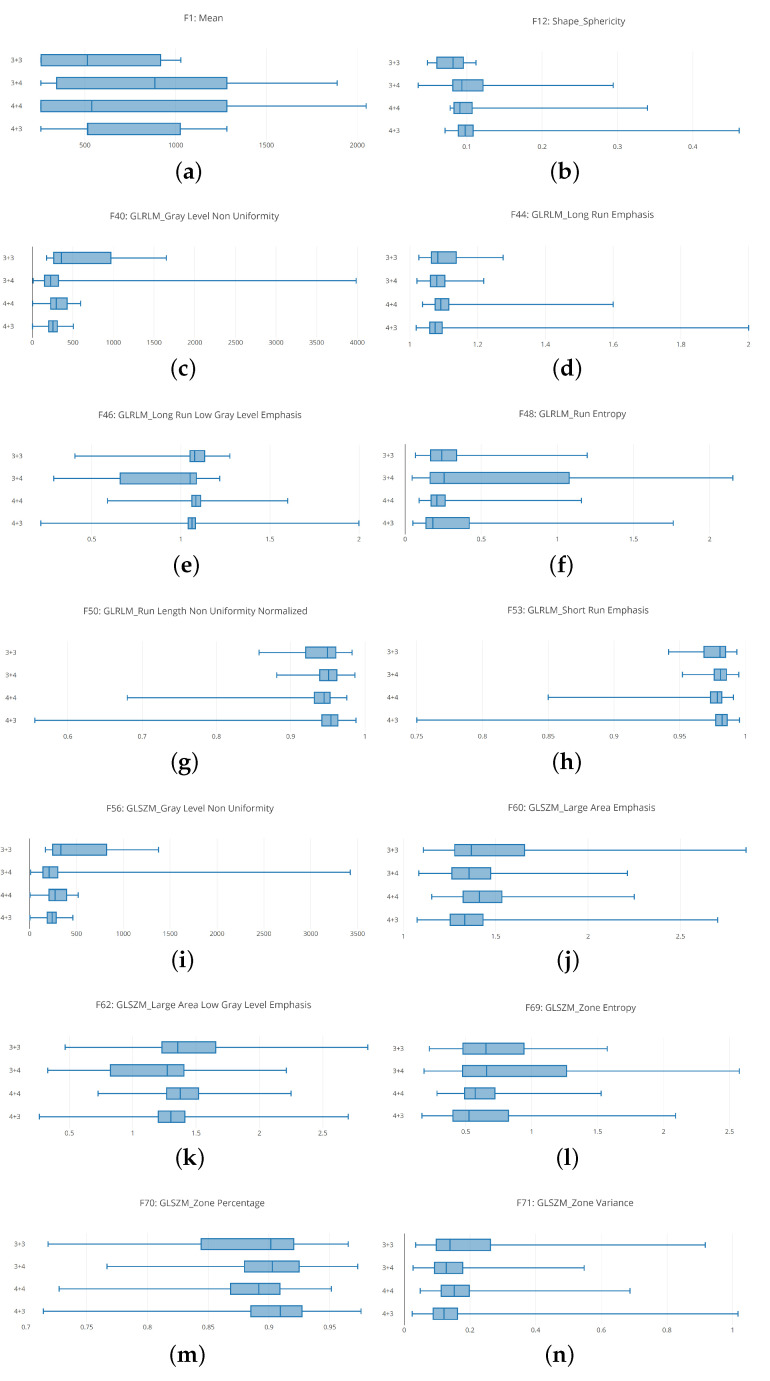
Descriptive Statistics: boxplots for a subset of the considered radiomic features (Mean (**a**), Shape Sphericity (**b**), Gray Level Non Uniformity (**c**), Long Run Emphasis (**d**), Long Run Low Gray Level Emphasis (**e**), Run Entropy (**f**), Run Length Non Uniformity Normalized (**g**), Large Area Emphasis (**h**), Gray Level Non Uniformity (**i**), Large Area Emphasis (**j**), Large Area Low Gray Level Emphasis (**k**), Zone Entropy (**l**), Zone Percentage (**m**), Zone Variance (**n**)).

The boxplot analysis (shown in Figure 5) suggests that distributions belonging to different Gleason score exhibit different values, symptomatic that the radiomic feature can be useful for discriminating between different Gleason score MRIs.

In detail, in Figure 5 are depicted boxplots related to 12 radiomic features.

Figure 5a shows the boxplots related to the unique First Order feature we consider i.e., the mean. It appears that the distribution of 4 + 3 Gleason score MRIs is thin if compared with the other ones. With regard to the 3 + 4 and 4 + 4 distributions they are similar (even if the medians of the 3 + 4 and 4 + 4 distribution is different), while the 3 + 3 distribution the smallest.

Figure 5b shows the boxplots related to the Shape Sphericity feature. The 4 + 3 and 4 + 4 boxplots are thinner if compared with the 3 + 3 and 3 + 4 ones. The widest is the one related to the distribution of the 3 + 4 Gleason score (the 3 + 3 and the 3 + 4 boxplots are the most different from the Shape Sphericity feature point of view).

Figure 5c shows the boxplots related to the Gray Level Non Uniformity feature. The 3 + 3 boxplot is the more extended one, if compared with the others exhibited from the 3 + 4, 4 + 3 and 4 + 4 distributions. The boxplot exhibited from the 4 + 3 distribution is the thinner one, while the boxplot related to the 3 + 3 distribution is largest one.

Figure 5d shows the boxplots related to the Long Run Emphasis feature. The 3 + 3 boxplot is slightly wider if compared with the others exhibited from the 3 + 4, 4 + 3 and 4 + 4 distributions. The boxplots exhibited from 3 + 4, 4 + 3 and 4 + 4 distributions are similar from a length point of view, but they have a different median.

Figure 5e shows the distributions related to Long Run Low Gray Level Emphasis. In these boxplots, it appears that the 3 + 4 distribution is wider if compared with to the other ones. The second wider one is the 3 + 3 distribution, but more near the 4 + 3 and 4 + 4 distributions. In particular the smallest one is the distribution related to the 4 + 3 Gleason score: this is symptomatic that the values for the Long Run Low Gray Level Emphasis feature in the 4 + 3 Gleason score prostate cancer range in a small numeric interval.

The boxplots related to the Run Entropy feature are shown in Figure 5f. The 3 + 4 Gleason score distribution is wider if compared with the other ones. This is symptomatic this feature can be a good candidate to discriminate between this Gleason score and the remaining ones. As a matter of fact, the values that populate the other distributions are ranging in a smaller interval. In particular the 4 + 4 distribution is the smallest.

Figure 5g shows the boxplots related to the Run Length Non Uniformity Normalized radiomic feature. These boxplots are spanning in similar numeric intervals. The 3 + 3 distributions exhibit a range slightly wider if compared with the ones related to the other Gleason scores. In this boxplot, the median exhibited by the several distributions are different, this is the reason why the features can be considered a quite good candidate to discriminate between different Gleason score MRIs.

The Large Area Emphasis radiomic feature belonging to the GLSZM category is depicted in Figure 5h. Similary to the Run Lenght Non Uniformity Normalized feature boxplots (represented in Figure 5g), the 3 + 3 distributions is slightly wider if compared to the other ones, while the 3 + 4, 4 + 4 and 4 + 3 distributions exhibit values belonging to similar numeric intervals but with different medians.

Figure 5i shows the comparison between the 3 + 3, 3 + 3, 4 + 4 and 4 + 3 boxplots related to the Gray Level Non Uniformity radiomic feature. The 3 + 3 distributions are the wider one if compared to the other ones. The second wider one is the distribution exhibited by the 4 + 4 Gleason score MRIs, the third wider one is the one exhibited by the 3 + 4 Gleason score MRIs while the smallest one is the distribution belonging to the 4 + 3 Gleason score MRIs.

The distributions related to the Large Area Emphasis radiomic feature is shown in Figure 5j. All the distributions span in a quite extended numeric interval (for instance, the boxplots are wider if compared to the ones depicted for the Short Run Emphasis feature in Figure 5h and the Gray Level Non Uniformity feature in Figure 5i). In detail, the 3 + 3 distribution is the wider one, while the 3 + 4 and 4 + 4 distributions exhibit similar extension, but the minimum in the 3 + 4 is lower if compared to the one related to the 4 + 4 distribution: in fact, the 3 + 4 distribution starts from a lower point with respect to the 4 + 4 distribution, while the higher point is exhibiting a similar value (in fact the two distributions end at a similar point). The 4 + 3 distribution is similar for the extension to the 3 + 4 one, but it is less wide if compared to the 3 + 4 one.

The boxplots related to the Large Area Low Gray Level Emphasis radiomic feature belonging to the GLSZM category are shown in Figure 5k. The wider distribution in the one belonging to the 3 + 4 Gleason score MRIs: this is symptomatic of the fact that this feature can be a good candidate to discriminate between 3 + 4 Gleason score prostate cancer MRIs and the other ones. The second wider distribution is the related to the 3 + 3 Gleason score MRIs, while the 4 + 4 and the 4 + 3 exhibit a similar extension, whit the difference that the minimum of the 4 + 4 distribution is higher with respect to the one of the 4 + 3 distribution, and the maximum of the 4 + 4 distribution is lower with respect to the one obtained from the 4 + 3 distribution.

Figure 5l shows the distributions related to the Zone Entropy radiomic feature belonging to the GLSZM category. Similarly to the distributions related to Large Area Low Gray Level Emphasis (shown in Figure 5k), the 3 + 4 boxplot is the wider one if compared to the other distributions: also this feature represents a good candidate to discriminate between Gleason score 3 + 4 MRIs and the other ones. With regard to the other distributions, the 3 + 3 one is the second widest one, while the 4 + 4 the third widest one. The 4 + 4 distribution is the smallest one.

Figure 5m shows the distributions related to the Zone Percentage radiomic feature belonging to the GLSZM category. The 3 + 3 distribution in the wider one, while the 3 + 4, 4 + 4 and 4 + 3 distributions exhibit a similar span. The difference between these three distributions is in the starting and ending point: as a matter of fact, the 4 + 3 distribution exhibits the lower minimum value, while the higher minimum value is obtained from the 3 + 4 distribution. From the higher value point of view, the maximum value is exhibited from the 4 + 3 distribution, while the lower one is obtained to the 4 + 4 distribution. The median of the four distributions is different.

The distributions for the Zone Variance feature belonging to the GLSZM category is depicted in Figure 5n. The 3 + 3 distribution is the wider one, while the 3 + 4, 4 + 4 and 4 + 3 distributions exhibit a similar extension. In detail the 3 + 4 and the 4 + 3 distributions are quite similar (with the difference that the maximum value of the 4 + 3 distribution is higher if compared to the one exhibited from the 3 + 4 distribution). With regard to the 4 + 4 distribution, its numeric interval range is populated by a set of numbers, which are higher if compared to the ones related to the 3 + 4 and 4 + 3 distributions.

**Remark** **1.** *From the descriptive statistics, we find that the distributions related to 3 + 3, 3 + 4, 4 + 3 and 4 + 4 Gleason score prostate MRIs range in different interval for the considered radiomic features. This may reveal that the features can be a good candidate for building a model with a good Gleason score prediction performance. Clearly, this result can be confirmed only by the hypotheses testing and by the classification analysis outcomes.*

### 3.2. Hypothesis Testing

The idea behind the hypothesis testing is to understand if the radiomic features exhibit different distributions for the patient afflicted by different Gleason score grade with statistical evidence.

We consider valid the results when both the considered tests reject the null hypothesis, which results is shown in Table 1.

For the features #15, #16, #21, #27, #28, #43, #44, #56, #66, while the Mann-Whitney test is not passed by features #16, #21, #27, #28, #43, #44 and #66 the Wald-Wolfowitz test is not passed. The #16, #21, #27, #28, #43, #44 and #66 features not passed both the Wald-Wolfowitz test and the Mann-Whitney one.

To conclude, the features that have not passed the null hypothesis $H_{0}$ test are the following: #1, #2, #3, #4, #5, #6, #7, #8, #9, #10, #11, #12, #13, #17, #18, #19, #20, #22, #23, #24, #25, #26, #29, #30, #31, #32, #33, #34, #35, #36, #37, #38, #39, #40, #41, #42, #45, #46, #47, #48, #49, #50, #51, #52, #53, #54, #55, #57, #58, #59, #60, #61, #62, #63, #64, #65, #67, #68, #70 and #71 i.e., 60 features on the 71 considered in the study.

**Remark** **2.** *The radiomic feature distributions show a statistically significant difference by running both the tests. In particular, 59 features on 71 passed the Mann–Whitney test and the Kolmogorov–Smirnov tests. The classification analysis with the designed deep neural network will confirm if the considered radiomic features are able to discriminate between prostate cancer MRIs labeled with different Gleason scores.*

### 3.3. Classification Analysis

In this section, we present the results of the experiment aimed to verify whether the designed deep neural network is able to discriminate between prostate cancer MRIs labeled with different Gleason score.

We considered the following metrics in order to evaluate the results of the classification: Sensitivity, Specificity, Accuracy and Loss.

The deep learning network training, with which a number of epochs equal to 10, is performed by splitting the dataset in two sub-dataset with an equal percentage of instances belonging to the four considered classes: 80% of the instances belonging to the 3 + 3, 3 + 4, 4 + 3 and 4 + 4 Gleason scores were included into the training dataset, while the remaining 20% were included in the testing dataset. We performed five different classifications, in order to evaluate the full dataset (we performed a five-fold cross validation).

The deep learning network training is performed by splitting the D#1 dataset into two sub datasets with an equal percentage of instances belonging to the four considered classes (i.e., GS): 80% of the instances belonging to the 3 + 3, 3 + 4, 4 + 3 and 4 + 4 classes into the training data-set (i.e., TD#1), while the remaining 20% was included in the testing dataset (i.e., ED#1). We performed five different classifications, in order to evaluate the full dataset (we performed a five-fold cross-validation). The full D#2 data-set (i.e., ED#2) is considered as the testing set. We consider the D#1 data-set for model training and for cross-validation, while the D#2 data-set is fully considered for the testing with the aim to evaluate the performances of the proposed method with data-set belonging to different institutions. In fact, the D#1 dataset was gathered from the Boston Medical Center, while the D#2 one from the National Cancer Institute in the USA. Table 2 shows the number of MRI slices considered for each Gleason score and the normal cases for the training and the testing of the proposed network, while Table 3 shows the performances obtained by the proposed deep neural network in Gleason score and normal cases prediction. The 300 slices belonging to the 10 patients normal cases, belonging to the third data-set, have been distributed in the following way: 200 slices in the TD#1 data-set, 50 slices in the ED#1 and the remaining 50 slices in the ED#2 data-set.

As shown in Table 3, the proposed method obtains a sensitivity ranging from 0.96 to 0.98, a specificity ranging from 0.97 to 0.99 and accuracy from 0.96 to 0.98 in Gleason score prediction. A sensitivity equal to 0.96, a specificity equal to 0.97 and an accuracy of 0.96 are reached in normal cases identification, confirming that the proposed deep learning network is able to discriminate between different Gleason scores, but also from prostate cancerous and healthy areas.

**Figure 6:**
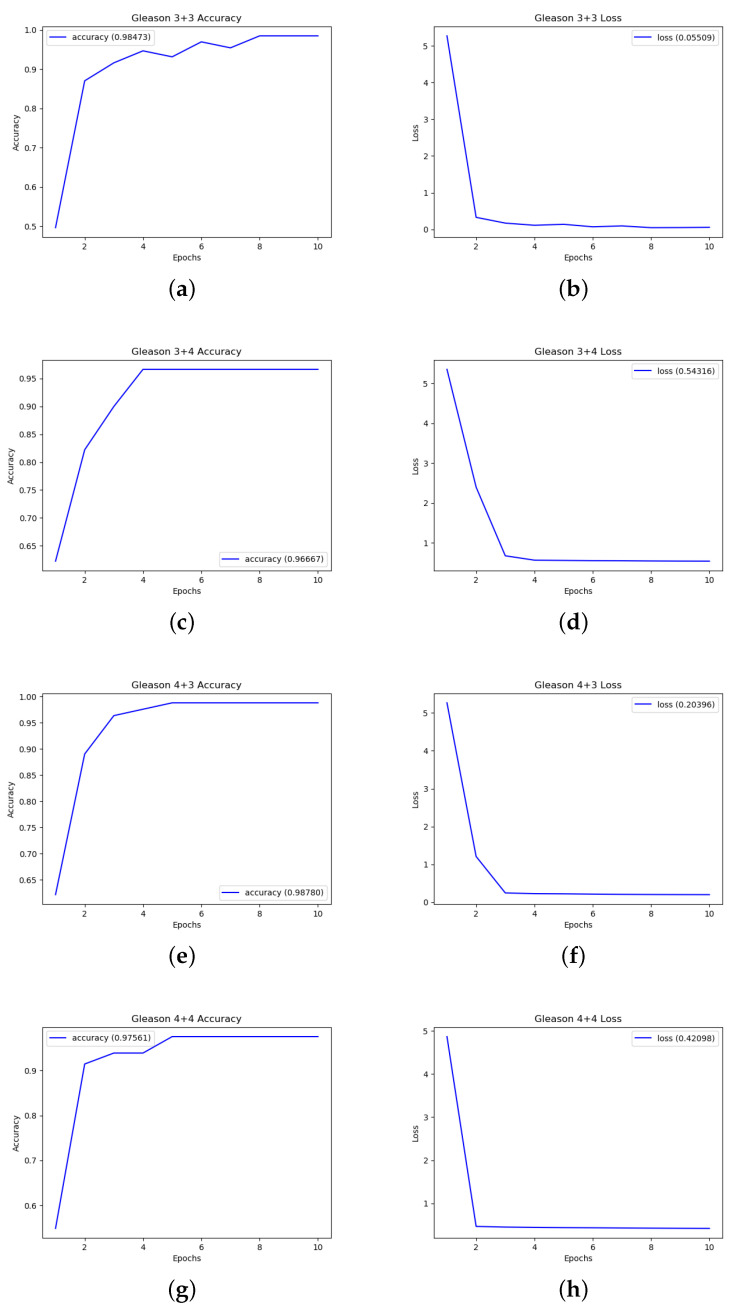
Classification analysis: results in terms in terms of accuracy (shown in (**a**) for the 3 + 3 Gleason score, (**c**) for the 3 + 4 Gleason score, (**e**) for the 4 + 3 Gleason score and (**g**) for the 4 + 3 Gleason score) and loss (shown in (**b**) for the 3 + 3 Gleason score, (**d**) for the 3 + 4 Gleason score, (**f**) for the 4 + 3 Gleason score and (**h**) for the 4 + 3 Gleason score).

Figure 6 shows the Accuracy and Loss plots.

Figure 6a,b shows, respectively, accuracy and the loss obtained for the 3 + 3 Gleason score prediction. The average accuracy is equal to 0.98473, while the average Loss is equal to 0.05509 considering 10 epochs. From the plots in Figure 6a,b the deep neural network reaches the lowest (highest) Loss (Accuracy) values after eight epochs: as a matter of fact, in the 9th and 10th epochs there is not Loss worsening (Accuracy improvement).

The performances obtained for 3 + 4 Gleason score prediction are shown in Figure 6c,d. The average accuracy is equal to 0.96667 and the average loss is equal to 0.54316 in the 10 considered epochs. Differently from the 3 + 3 plots (depicted in Figure 6a,b), after four epochs the deep neural network reaches the best tuning.

Plots in Figure 6e,f are related to the performances obtained for the Gleason 4 + 3 prediction. The deep neural network obtains an accuracy equal to 0.98780 and a loss equal to 0.20396 on the 10 epochs. The deep neural network employs three epochs to reach the best tuning.

Average accuracy and average loss related to the 4 + 4 Gleason score prediction are plotted in Figure 6g,h. An accuracy equal to 0.97561 and a loss equal to 0.42098 is reached by the deep neural network. The number of epochs employed to reach the lowest (highest) loss (accuracy) values are equal to 5.

Furthermore, to demonstrate the effectiveness of the designed deep network in Gleason score detection, we performed the experiment with supervised machine learning algorithms: J48, RandomForest (RF), Bayesian Network (BN), Neural Network (NN), Support Vector Machine (SVM) and K-nearest neighbors (kNN) with a five-cross fold validation (i.e., the same folds involved in the evaluation of the deep learning network). The difference between the proposed deep learning network and the NN algorithm relies on the architecture: as a matter of fact, the neural network in the machine learning experiment is very simple considering that contains only one hidden layer. From the other side, the deep learning network we propose is composed of four 1D convolutional layers, one pooling layer, one flatten layer, one dropout Layer and one dense layer.

The supervised machine learning algorithms take into account with following parameters:

- J48: bathSize equal to 100, confidence factor of 0.25:
- RF: bathSize 100, bagSizePercent 100, numIterations 100;
- BN: bathSize 100, maxParents 1.
- NN: epoch 10, batchsize of 100,
- SVM: kernel function degree equal to 3, batchsize of 100,
- kNN nearest neighbours equal to 1, batchsize of 100.

Table 4 shows the machine learning results in Gleason score detection.

As shown by the results in Table 4, the best results are obtained by the SVM model with an accuracy ranging from 0.85 (3+4 Gleason score), 0.86 (3+3 Gleason score), 0.87 (4+3 and 4+4 Gleason score) and 0.88 for the normal cases detection.

Moreover, as additional comparison to show the radiomic features effectiveness, we consider the Embedded Localisation Features (ELF) [20], aimed at extracting the feature locations from a pre-trained network with the aim to build a detector, by avoiding hand-crafted feature detection. This information is computed from the gradient of the feature map with respect to the input image. This provides a saliency map with local maxima on relevant keypoint locations. In particular we consider as a pre-trained network the VGG model, a convolutional neural network achieving 92.7% top-5 test accuracy in the ImageNet dataset evaluation, composed of over 14 million images belonging to 1000 different classes. ELF requires neither supervised training nor fine-tuning and it is considered as preprocessing with the aim to compare the features obtained by exploiting ELF with the considered radiomic features. As stated from authors in [20] high-level feature maps usually exhibit a wider receptive field hence take higher context into account for the description of a pixel location. This leads to more informative descriptors which motivate us to favor higher level maps. As a matter of fact, whether the feature map level is too high, the interpolation of the descriptors generate vectors too similar to each other. For instance, the VGG pool4 layer produces more discriminative descriptors than pool5 even though pool5 embeds information with higher-level semantics. We experiment on the VGG pool4 layer considering the task of discrimination between different Gleason score and normal patients. Authors in [20] provide the code repository freely available for research purposes (https://github.com/abenbihi/elf) Results of this experiment are shown in Table 5.

As shown from the ELF experiment result, it emerges that the method proposed in [20] obtains an accuracy ranging from 0.94 (for the normal patient detection) to 0.97 (for the 3 + 3 Gleason score detection), overcoming the performances reached from the machine learning experiment: as a matter of fact the best performances reached in the supervised machine learning experiment are the SVM one with an accuracy ranging from 0.85 (for the 3 + 4 Gleason score detection) to 0.88 (for the normal patient detection). We highlight that the ELF method does not overcome the proposed method, based on the adoption of radiomic features: as a matter of fact, we obtain slightly higher accuracy, from 0.96 for normal patient detection to 0.98 for 3 + 4 and 4 + 3 Gleason score detection. We think that the proposed method obtains higher accuracy because the area of interest (i.e., the cancerous one) is selected by radiologist and it is not gathered by a model.

Below we show the performances obtained by the proposed method in patient classification. As a matter of fact the proposed method considers a single slice as an instance, and for this reason we compute the sensitivity, the specificity and the accuracy metrics for each patient, with the aim to provide to radiologists a diagnostic tool.

In particular, we apply the following formula for the patient-grain performance:

$$PP\;=\;\frac{\#MRI_{t}}{\#MRI},$$

where PP stands for Patient Prediction, $\#MRI_{t}$ represents the number of MRI predicted with the right Gleason score and #MRI is the total number of MRI belonging to the patient under analysis.

We mark a patient as rightly predicted if PP ≥ 0.5.

In Table 6 we show the results obtained for the patient-grain experiment.

This analysis is confirming the results previously obtained: the accuracy is ranging from 0.96 to 0.98 even for the patient-grain experiment.

Considering the importance in the medical context to reduce the time and effort for the analysis we report below the time performance analysis.

With respect to computational time the proposed method for training employed approximately 24 min for training and 5 min for testing. With regard to the supervised machine learning experiments, in average the machine learning models employed 6 min for training and 2 min for testing.

The time employed to make a prediction for a patient under analysis is approximately 27 s. The experiments were performed on a Microsoft Windows 10 machine equipped with the following configuration: 8th Generation Intel Core i7 2.0 GHz CPU, NVIDIA GeForce MX150 graphic card, 16 GB RAM memory and 500 GB Hard Disk.

Remark 3: The classification analysis suggests that the developed deep neural network input with the considered radiomic features is able to discriminate between different Gleason score prostate cancer MRIs with encouraging performances. As a matter of fact the Accuracy obtained is equal to 0.98473 for the Gleason 3 + 3 detection, 0.96667 for the Gleason 3 + 4 detection, 0.98780 for the Gleason 4 + 3 detection and 0.97561 for the Gleason 4 + 4 detection.

## 4. Discussion

As discussed in the Introduction section, the aim of the following work is the Gleason score prediction through a deep neural architecture exploiting a set of radiomic features.

In this section, we propose a reasoned discussion about the current state-of-the-art literature related to Gleason score prediction. First, we describe related literature aimed to detect the Gleason score, and then we focus on papers exploiting radiomic features. For each related work we highlight the main differences with our proposed method.

### 4.1. Prostate Cancer Prediction

Tabesh et alius [21] present a study of image features for cancer diagnosis and Gleason grading starting from the histological images of the prostate. Differently from our proposal, this method can be considered invasive: as a matter of fact, the method we propose requires only MRIs, not an invasive analysis to obtain the imaged tissue. They consider as features aggregate color, texture, and morphometric cues at the global and histological object levels for the classification task, while on the other side we consider a set of 71 radiomic features. They consider state-of-the-art classification algorithms (i.e., Gaussian, k-nearest neighbor, and support vector machine) obtaining an accuracy equal to 81% into low- and high-grade classes, while we propose a deep neural network aimed to discriminate between the several Gleason score (not only the low- and high-grade classes): the accuracy we obtain, as described in the evaluation results section, is ranging from 96% to 98%.

Arvaniti and colleagues [22] analyse tissue microarrays for Gleason score prediction. The first difference with the method we propose is the way in which authors obtain the features: they consider tissue biopsies to gather their features, therefore the patient must undergo biopsy and for this reason, it is considered an invasive method. Differently, we consider a set of radiomic features directly obtained from MRIs.

They consider pre-trained deep learning widespread models for image recognition i.e., VGG-16, Inception V3, ResNet-50, MobileNet and DenseNet-121. These networks are included in the TensorFlow APIs. From the other side, we define and implement the deep neural network from the scratch. From their experiment, it emerges that stromal regions were wrongly predicted as belonging to Gleason pattern 3.

Kumar et alius [23] also consider tissue images to detect prostate cancer through deep learning. They obtain an accuracy equal to 0.81 considering three convolutional layers, while in the deep neural network we designed we consider four convolutional layers (with the aim to extract more complex features) obtaining an accuracy ranging from 0.96 to 0.98 using radiomic features. Furthermore, they consider a dataset of tissue images marked as cancer or not cancer: for this reason their method is able to label a tissue image as cancerous or not cancerous. The method we propose considers several prostate cancer stages by detecting the Gleason score in order to know the disease progression.

Authors in [24] consider a modified version of the Inception V3 model (a deep learning architecture) to identify prostate cancers. The obtain an accuracy equal to 0.70. The main differences with the method we propose are the following: we consider a set of radiomics features, we designed the deep architecture to build the model and the better obtained performances.

Researchers in [25] experiment three different state-of-the-art supervised machine learning algorithms with the aim to identify several types of cancer using genes expression as feature vector. With regard to prostate cancer they obtain the following results in terms of accuracy: 67.65, 73.53 and 67.65 respectively using the C4.5 decision tree, bagged and boosted decision tree. Differently, through the adoption of radiomic features, the deep neural network we developed is able to reach better performances in terms of accuracy. Furthermore, their prediction is binary (i.e., cancer/no cancer), while the deep architecture we propose is able to detect the stage reached by the prostate cancer.

### 4.2. Prostate Cancer Prediction Using Radiomic Features

Hussain and colleagues [26] propose a method to identify prostate cancer using a Bayesian network. They start from a set of MRI images related to Prostate and Brachytherapy. The main difference with our method is that the deep neural network we designed is able to discriminate between several Gleason scores prostate cancers. The first difference with the method we propose is from the methodology point of view: as a matter of fact, we designed a deep neural network, we do not apply well-known machine learning algorithms. The second is related to the detection target, authors in this work did not consider the different stages of prostate cancer (their classification is binary:cancer/no cancer), while the method we propose is aimed to discriminate between several Gleason scores prostate cancers.

Authors in [27] exploit a set composed of five different features. They perform a statistical analysis with the considered features to discriminate from different Gleason grades obtaining a p<0.05. In this research, authors show that texture features are not of interest for Gleason grade detection. Differently from the proposed approach, Chaddad et al. in [27] basically propose a statistical analysis: authors do not consider the Gleason grade prediction starting from MRIs. Moreover, they exploit five radiomic features, while we take into account an extended set composed by 71 different radiomic feature.

Khalvati and colleagues [28] analyze radiomics texture feature models (composed of 96 different features) to detect prostate cancer in MRI images. They obtain a sensitivity equal to 0.86 in binary labeling an MRI under analysis in the tumor (considering only tumors related to a Gleason score of seven and above) or benign. The first different with the method we propose is related to a finer grain detection, as a matter of fact, we detect the cancer stages (i.e., the several Gleason scores), the second one is related to the proposal of a novel developed by authors deep architecture and, as third point, the performances obtained that with regard to the method we propose are ranging from 96% to 98%.

Authors in [29] investigate prostate lesions considering different MR techniques i.e., T1-weighted and T2-weighted. Their main outcome is that T2 values alone achieves a diagnostic accuracy of 0.85 and exhibits an improved discriminating performance of 0.89, when combined with DCE T1-w features. Differently, considering that we propose a deep learning architecture, the feature selection process is automatically performed by the hidden states of the network. Furthermore, we consider a more extended feature set of 71 radiomic features.

Artificial intelligence technique i.e., machine learning is exploited by authors in [30], where a model is built with texture features with a prediction accuracy equal to 0.88. Researchers in [30] adopt the Bayesian classifier, differently the proposed approach overcomes the accuracy of well-known machine learning techniques by employing deep learning.

Authors in [31] exploit supervised machine learning to detect patients afflicted with prostate cancer. By exploiting features related to volume, age and prostate-specific indicators they obtain a prostate cancer detection rate equal to 86.6%. They differentiating prostate cancer from benign prostatichyperplasia without considering the different prostate cancer stages, differently from the deep learning-based method we propose.

Researchers in [32] adopt an approach based on deep learning to distinguish from high and low-grade localized cancers. In the evaluation, they obtain an accuracy of 0.7. They do not provide details about the considered architecture. The method we propose is aimed to detect also prostate tumors from the low and the low-grade (for example the cancers marked with the 3 + 4 and the 4 + 3 Gleason grades).

Authors in [33] proposed a method for the detection of prostate tumors employing a supervised machine learning algorithm. Their feature vector is composed of features gathered from nuclei. Their model reaches an accuracy of 0.83. With respect to the proposed method, the proposed architecture reaches an accuracy ranging between 0.96 and 0.98, obtaining better performances.

Authors in [34] propose an approach based and the random forest classification algorithm for multiview learning. They consider radiomic features by studying the effects of hyperparameters on the quality of random forest dissimilarity.

Researchers in [35] exploit a model, based on the shape and gray-level radiomic features for therapy decision support related to metastases prediction on lung cancer. They consider three machine learning classifiers used up to 100 selected features to perform the analysis.

As it emerges from the review of current literature related to the application of machine and deep learning techniques to prostate cancer detection, this represents the first tentative to discriminate between the several types of prostate cancers according to Gleason score through deep learning techniques exploiting radiomic features.

## 5. Conclusions

The MRI labeling activity from the radiologist point of view is a crucial and time-consuming human task. In this paper we proposed a method aimed to automatically classify an MRI prostate cancer assigning the correct Gleason score. We considered a radiomic feature set of 71 features belonging to five different categories: First Order, Shape, Gray Level Co-occurrence Matrix, Gray Level Run Length Matrix and Gray Level Size Zone Matrix. The designed deep learning network considers several convolutional layers aimed to extract more abstract and deep information from the network. We considered also two additional layers, a Flatten and a Dropout one, in order to remove the possible network overfitting. We obtain an accuracy for Gleason score prediction equal to 0.98473, 0.96667, 0.98780 and 0.97561 with regard to Gleason score 3 + 3, Gleason score 3 + 4, Gleason score 4 + 3 and Gleason score 4 + 4 prediction, respectively. Moreover, the proposed model is also able to discriminate between prostate cancerous and healthy areas. Three public available MRIs dataset labeled by radiologists has been used for replication purposes.

As future work, we plan to evaluate the proposed method using MRIs belonging to different diseases (for instance, brain tumor). As a matter of fact one of the weaknesses afflicting model data-driven is the generability: for this reason, we aim to investigate whether it is possible to generate a model able to detect different kinds of tumor grades. Furthermore, we want to investigate whether formal verification techniques, for instance model checking or deductive verification, can be useful to overcome the obtained accuracy. Another interesting research direction is represented by the automatic detection of different disease grades without the need from the radiologist to mark the ROI, as a matter of fact, this list of future work can help for reducing the time and the effort by fully automatizing the analysis.

## Figures and Tables

**Table 1:** Hypothesis testing: results of the null hypothesis $H_{0}$ test.

# Feature	Wald–Wolfowitz	Mann–Whitney	Test Result
[1–8]	*p* < 0.001	*p* < 0.001	passed
9	*p* < 0.001	*p* < 0.001	passed
[10–14]	*p* < 0.001	*p* < 0.001	passed
15	*p* > 0.10	*p* < 0.001	not passed
16	*p* > 0.10	*p* > 0.10	not passed
[17–19]	*p* < 0.001	*p* < 0.001	passed
20	*p* < 0.001	*p* < 0.001	passed
21	*p* > 0.10	*p* > 0.10	not passed
22	*p* < 0.001	*p* < 0.001	passed
23	*p* < 0.001	*p* < 0.001	passed
24	*p* < 0.001	*p* < 0.001	passed
25	*p* < 0.001	*p* < 0.001	passed
26	*p* < 0.001	*p* < 0.001	passed
[27–28]	*p* > 0.10	*p* > 0.10	not passed
29	*p* < 0.001	*p* < 0.001	passed
30	*p* < 0.001	*p* < 0.001	passed
31	*p* < 0.001	*p* < 0.001	passed
32	*p* < 0.001	*p* < 0.001	passed
[33–36]	*p* < 0.001	*p* < 0.001	passed
37	*p* < 0.001	*p* < 0.001	passed
38	*p* < 0.001	*p* < 0.001	passed
39	*p* < 0.001	*p* < 0.001	passed
40	*p* < 0.001	*p* < 0.001	passed
[41–42]	*p* < 0.001	*p* < 0.001	passed
43	*p* > 0.10	*p* > 0.10	not passed
44	*p* > 0.10	*p* > 0.10	not passed
45	*p* < 0.001	*p* < 0.001	passed
46	*p* < 0.001	*p* < 0.001	passed
47	*p* < 0.001	*p* < 0.001	passed
48	*p* < 0.001	*p* < 0.001	passed
49	*p* < 0.001	*p* < 0.001	passed
50	*p* < 0.001	*p* < 0.001	passed
51	*p* < 0.001	*p* < 0.001	passed
52	*p* < 0.001	*p* < 0.001	passed
53	*p* < 0.001	*p* < 0.001	passed
54	*p* < 0.001	*p* < 0.001	passed
55	*p* < 0.001	*p* < 0.001	passed
56	*p* > 0.10	*p* < 0.001	not passed
57	*p* < 0.001	*p* < 0.001	passed
[58–62]	*p* < 0.001	*p* < 0.001	passed
63	*p* < 0.001	*p* < 0.001	passed
64	*p* < 0.001	*p* < 0.001	passed
65	*p* < 0.001	*p* < 0.001	passed
66	*p* > 0.10	*p* > 0.10	not passed
67	*p* < 0.001	*p* < 0.001	passed
68	*p* < 0.001	*p* < 0.001	passed
69	*p* < 0.001	*p* < 0.001	passed
70	*p* < 0.001	*p* < 0.001	passed
71	*p* < 0.001	*p* < 0.001	passed

**Table 2:** The datasets.

Label	TD#1	ED#1	ED#2	Tot
3+3	123	31	78	232
3+4	270	68	234	572
4+3	138	35	182	355
4+4	127	32	182	341
normal	200	50	50	300

**Table 3:** Performance evaluation.

Label	Sens.	Spec.	Acc.
3+3	0.98	0.98	0.98
3+4	0.96	0.97	0.96
4+3	0.98	0.99	0.98
4+4	0.97	0.98	0.97
normal	0.96	0.97	0.96

**Table 4:** Machine learning experiment.

Algorithm	Sensitivity	Specificity	Accuracy	Label
	0.76	0.77	0.76	3 + 3
	0.77	0.77	0.76	3 + 4
J48	0.76	0.75	0.76	4 + 3
	0.74	0.73	0.74	4 + 4
	0.72	0.72	0.73	normal
	0.78	0.079	0.79	3 + 3
	0.77	0.77	0.77	3 + 4
RF	0.76	0.77	0.76	4 + 3
	0.78	0.78	0.78	4 + 4
	0.75	0.76	0.75	normal
	0.76	0.77	0.76	3 + 3
	0.75	0.75	0.75	3 + 4
BN	0.75	0.76	0.75	4 + 3
	0.76	0.76	0.76	4 + 4
	0.77	0.76	0.77	normal
	0.80	0.79	0.80	3 + 3
	0.79	0.79	0.79	3 + 4
NN	0.80	0.81	0.80	4 + 3
	0.81	0.80	0.80	4 + 4
	0.82	0.80	0.81	normal
	0.85	0.84	0.86	3 + 3
	0.84	0.85	0.85	3 + 4
SVM	0.86	0.87	0.87	4 + 3
	0.87	0.86	0.87	4 + 4
	0.88	0.89	0.88	normal
	0.77	0.77	0.77	3 + 3
	0.75	0.74	0.74	3 + 4
kNN	0.79	0.77	0.78	4 + 3
	0.79	0.77	0.79	4 + 4
	0.77	0.77	0.78	normal

**Table 5:** Embedded Localisation Features (ELF) experiment.

Sensitivity	Specificity	Accuracy	Label
0.96	0.96	0.97	3 + 3
0.96	0.95	0.95	3 + 4
0.96	0.98	0.97	4 + 3
0.94	0.95	0.96	4 + 4
0.94	0.95	0.94	normal

**Table 6:** Patient-grain experiment.

Sensitivity	Specificity	Accuracy	Label
0.96	0.98	0.97	3 + 3
0.95	0.96	0.96	3 + 4
0.97	0.98	0.98	4 + 3
0.96	0.96	0.96	4 + 4
0.96	0.97	0.96	normal
